# Diabetes and Alzheimer’s Disease: Might Mitochondrial Dysfunction Help Deciphering the Common Path?

**DOI:** 10.3390/antiox10081257

**Published:** 2021-08-06

**Authors:** Maria Assunta Potenza, Luca Sgarra, Vanessa Desantis, Carmela Nacci, Monica Montagnani

**Affiliations:** Department of Biomedical Sciences and Human Oncology-Section of Pharmacology, Medical School, University of Bari “Aldo Moro”, Polyclinic University Hospital of Bari, p.zza G. Cesare 11, 70124 Bari, Italy; sgarraluca@gmail.com (L.S.); vanessa.desantis@uniba.it (V.D.); carmela.nacci@uniba.it (C.N.)

**Keywords:** mitochondrial dysfunction, type 2 diabetes (T2DM), Alzheimer’s disease (AD)

## Abstract

A growing number of clinical and epidemiological studies support the hypothesis of a tight correlation between type 2 diabetes mellitus (T2DM) and the development risk of Alzheimer’s disease (AD). Indeed, the proposed definition of Alzheimer’s disease as type 3 diabetes (T3D) underlines the key role played by deranged insulin signaling to accumulation of aggregated amyloid beta (Aβ) peptides in the senile plaques of the brain. Metabolic disturbances such as hyperglycemia, peripheral hyperinsulinemia, dysregulated lipid metabolism, and chronic inflammation associated with T2DM are responsible for an inefficient transport of insulin to the brain, producing a neuronal insulin resistance that triggers an enhanced production and deposition of Aβ and concomitantly contributes to impairment in the micro-tubule-associated protein Tau, leading to neural degeneration and cognitive decline. Furthermore, the reduced antioxidant capacity observed in T2DM patients, together with the impairment of cerebral glucose metabolism and the decreased performance of mitochondrial activity, suggests the existence of a relationship between oxidative damage, mitochondrial impairment, and cognitive dysfunction that could further reinforce the common pathophysiology of T2DM and AD. In this review, we discuss the molecular mechanisms by which insulin-signaling dysregulation in T2DM can contribute to the pathogenesis and progression of AD, deepening the analysis of complex mechanisms involved in reactive oxygen species (ROS) production under oxidative stress and their possible influence in AD and T2DM. In addition, the role of current therapies as tools for prevention or treatment of damage induced by oxidative stress in T2DM and AD will be debated.

## 1. Introduction

Diabetes mellitus (DM) is a chronic metabolic disorder characterized by hyperglycemia and a wide spectrum of complications including cardiovascular, ocular, renal, and immunological disturbances. In recent years, more and more attention has been focused on diabetes-related neurological deterioration, represented by a progressive impairment of cognitive abilities [1,2]. Currently, more than four hundred million people worldwide are affected by diabetes and this number is expected to increase dramatically by the next 30 years [3]. There are two major sub-forms of DM: insulin-dependent type 1 DM (T1DM) and not insulin-dependent type 2 DM (T2DM); this last accounts for approximately 90% of all cases of diabetes. The overt hyperglycemic condition in T2DM develops when a reduced responsiveness to insulin in peripheral target tissues, such as skeletal muscle, adipocytes, and liver, cannot be compensated with the adequate secretion of insulin by pancreatic beta-cells [4]. The effects of T2DM on the brain structure and function are currently well recognized, and clinical and epidemiological studies have shown that in patients with T2DM the risk of developing Alzheimer’s disease (AD) is twice that of non-diabetic individuals [5,6]. Both hyperglycemia and hyperinsulinemia may trigger neuronal death followed by neurodegenerative disease [7], and both may represent a risk factor for cognitive decline and AD, even before overt diabetes development [8]. Indeed, in the natural history of T2DM, the interaction of key genes with environmental factors such as physical inactivity, quality and quantity of nutrients, and aging concur to promote adiposity, impair β-cell function and reduce insulin effectiveness. When pancreatic insulin secretion is no longer sufficient to compensate for insulin resistance, glucose intolerance progresses to chronic hyperglycemia and overt diabetes. This implies that the condition of insulin resistance usually precedes by many years the onset of T2DM [9]. Insulin is the main glucoregulatory hormone in both peripheral and central nervous system (CNS): by activating its tyrosine kinase membrane receptor (IR), insulin contributes to body energy homeostasis, modulates the synaptic plasticity and cognition, and, when it does not work properly, is involved in aging-related neurodegeneration [10]. The notion that the brain consumes about 18–30% of total body glucose underlines the thigh link between physiological regulation of glucose uptake and proper brain function: indeed, in prediabetic patients, the increase of blood glucose levels is related to memory impairment [11]. On the same line, several studies indicate that older diabetic population is more susceptible to aging-associated cognitive decline (i.e., decreased executive functions, memory skills and processing speed) than aged individuals without diabetes [12]. 

AD is a neurodegenerative disorder representing the most common cause of dementia in the elderly; it usually starts slowly and worsens over time [13]. Over 48 million people worldwide are affected by AD or related dementias. Because of the increasing proportion of older people in the overall human population, it is predicted that by 2050 more than 140 million people worldwide will suffer from AD [14]. The earliest symptom of AD is short-term memory loss, followed later, as the disease progresses, by language difficulties, disorientation, mood swings, loss of motivation, inability to manage self-care and behavioral issues [15]; the disease often culminates with the patient’s death 3–9 years after diagnosis [16]. The neuropathological features of AD are represented by the brain accumulation of extracellular senile plaques and fibrils composed by aggregated amyloid-β-peptides, intracellular neurofibrillary tangles (NFTs) mainly consisting of hyperphosphorylated tau protein, microglial infiltration, neuroinflammation and significant neuronal loss [17,18]. Epidemiologic studies in the elderly, as well as experimental investigations in humans and animal models, have consistently suggested that lower brain glucose uptake and dysfunctional brain insulin signaling, termed as “brain insulin resistance” promote and accelerate cognitive dysfunction and AD progression [19]. Indeed, AD has been proposed as “type 3 diabetes”, a form of diabetes that selectively involves the brain, with molecular and biochemical features that overlap with both T1DM and T2DM [20]. Although not officially recognized by the World Health Organization (WHO) or by the American Diabetes Association (ADA), the term type 3 diabetes underlines the tight connection between these two apparently distinct diseases. Consistent with this view, more than 80% of patients suffering from AD develop diabetes or glucose intolerance [21], and postmortem analysis of brain from AD patients has detected a significant decreased expression and activation of IR, insulin-like growth factor 1 (IGF-1) and insulin receptor substrate-1 (IRS-1) [22], with a pattern resembling that observed during age-related changes [23]. 

**Figure 1:**
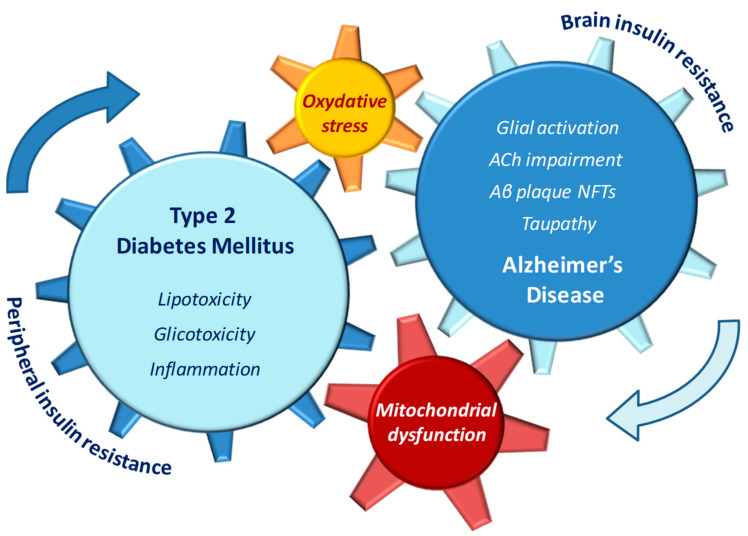
Pathophysiological features of type 2 diabetes (T2DM) and Alzheimer’s disease (AD) that may reciprocally influence and reinforce the progression of both diseases.

Insulin resistance has been repeatedly considered a direct causal factor for AD development, since a down-regulation in insulin signaling and a concomitant activation of stress kinases such as c-Jun-n-terminal kinases (JNK) are known to contribute to Aβ deposition and tau phosphorylation, followed by accumulation of NFT in the brain [24,25]. In addition, in T2DM patients, other AD-like brain changes linked with cognitive decline such as mitochondrial dysfunction [26,27], neuroinflammation, impaired learning and memory, and synaptic plasticity deficits [28] further support the presence of a causative link between diabetic and AD pathophysiology. Altered lipid metabolism and mitochondrial dysfunction may be among the molecular mechanisms underlying the increased risk of AD in diabetic patients [29]. Mitochondrial fatty acid oxidation is the source of cell energy metabolism and represents a key process in the maintenance of cellular lipid homeostasis [30]. During the progression of diabetes, any impairment in brain mitochondria electron chain may result in accumulation of fatty acid molecules and subsequent mitochondrial dysfunction. In turn, the increased levels of oxidative stress may trigger apoptotic death in neuronal cell [31,32] (Figure 1).

Mitochondrial dysfunction has therefore been proposed as a prominent and early oxidative stress-associated factor in aging and diabetes, and considered a key player in the enhanced susceptibility to neurodegenerative diseases, including AD [33]. On this respect, it is important to underline that the brain high energy requirements are strictly dependent on mitochondria activities, thus making the brain more susceptible to oxidative damage than other districts of the body. Based on these findings, the relationship between early mitochondrial dysfunction and the accumulation of Aβ in mitochondria has been regarded as one of the major determinants for energy failure, respiratory chain impairment, reactive oxygen species (ROS) generation, dysregulation of mitochondrial permeability transition pore (mPTP), imbalance of calcium homeostasis and even mitochondrial DNA/RNA mutations [34]. These observations suggest that among the multiple pathophysiological overlapping features in both T2DM and AD, mitochondrial dysfunction and oxidative stress represent the most relevant, thus pointing out to the mitochondrion as one fundamental target of scientific research [35].

A deeper understanding of mechanisms by which oxidative stress and mitochondrial dysfunction participate to the pathophysiology of T2DM and AD may be helpful to comprehend how these two seemingly unrelated diseases can exacerbate each other, and offer novel insights for potential therapeutic strategies aiming at modulating the onset and progression of both disorders. This review summarizes the main pathophysiological features of T2DM and AD that may reciprocally influence and reinforce the progression of both diseases, focusing on mitochondrial dysfunction as a unifying mechanism and a potential target for future preventative approaches.

## 2. Insulin-Signaling Impairment and Neurodegeneration 

Insulin exerts multiple anabolic activities via a complex signaling pathway regulating cell metabolism, cell growth, and cell differentiation. The main features of insulin-signaling pathways have been extensively described elsewhere [9]. Briefly, under physiological conditions, insulin binding to IR triggers the activation of interrelated intracellular cascades, mainly represented by the Ras/Raf/MEKK/MAPK pathways and the IRSs/PI3K/AKT pathways. Interestingly, the latter is deeply involved in downstream signaling network regulating protein synthesis, Aβ clearance and activity of glycogen synthase kinase-3 (GSK-3β). The role of GSK-3β in turn, is crucial for phosphorylation of tau, a soluble microtubule-binding protein whose physiological activity stabilizes microtubules in axons and contributes to neuronal growth, neuronal survival, synaptic plasticity and learning memory [24,36,37]. Moreover, as one key mediator of apoptosis, GSK-3β might directly contribute to neuronal loss in AD [38].

Although in peripheral tissues insulin stimulates glucose uptake into muscles and adipose tissues and inhibits hepatic gluconeogenesis during fed state [39], in the brain insulin is important for neuronal survival and synaptic plasticity and function [40]. Once considered an insulin-insensitive organ, the brain is currently recognized as a target for insulin action, and the IR density is particularly high in regions of the CNS such as the hippocampus, involved in memory [41], the hypothalamus, critical for metabolic control [42,43], as well as in other areas including the olfactory bulb, cerebellum, amygdala and cerebral cortex [44]. 

As a large peptide hormone, insulin cannot cross the blood–brain barrier (BBB) passively; however, insulin is found in the cerebrospinal fluid (CSF), therefore implying that peripherally produced insulin may reach the brain regions. Consistent with this, insulin levels in CSF—generally lower than in blood levels—tend to increase after meals or peripheral insulin infusion [45]. In some areas of the brain such as the hypothalamus, insulin access is facilitated by the lack of an effective BBB [46]. In other regions, the presence of insulin has been explained as a result of a saturable transcytosis process mediated by IR on vascular endothelium [47]. Insulin regulates the concentration of several neurotransmitters with essential roles in memory processes, such as acetylcholine (ACh), norepinephrine and epinephrine [48]; acting on both peripheral sites, as well as on brain areas, insulin controls the glucose metabolism and supports cholinergic functions involved in neuronal plasticity and neurogenesis, as well as in learning, memory, and myelin maintenance [49,50]. Thus, under physiological conditions, insulin acts as a protective factor for brain function and contributes to the prevention of cognitive decline. On the other hand, a dysregulated brain insulin signaling, defined as “brain insulin resistance”, has been proposed among factors responsible for AD progression [19], and the impaired activity of several mediators in the insulin-signaling pathways may contribute to neurodegeneration and AD symptoms [51,52].

As pointed out before, a clinical diagnosis of both vascular dementia and AD is up to 73% more frequent in patients with T2DM with respect to healthy subjects, and the process of cognitive decline seems to begin early in prediabetic stages of insulin resistance [53]. Consistent with the idea that vascular defects play a critical role in AD pathogenesis [54] the impaired endothelial and vascular function dependent on insulin defective signaling might also contribute, at least in part, to explain these observations [55,56]. The higher plasma insulin and lower CSF insulin found in AD patients compared to healthy adults support the hypothesis that a decreased insulin transport into the brain may trigger cognitive decline and neurodegeneration [57]. Although not all individuals with T2DM develop AD, and not all patients with dementia have diabetes [58], the correlation between both diseases is reinforced by an increasing number of shared pathophysiological features and common molecular mechanisms, such as amyloid peptide aggregation, inflammation, oxidative stress and mitochondrial dysfunction [59,60,61]. The following paragraphs briefly recapitulate the main findings connecting each of these abnormalities to T2DM and AD. 

### 2.1. Amyloidogenic Links between T2DM and AD

It has long been suggested that amyloidogenesis, a condition in which a soluble protein turns into insoluble fibrillar protein aggregates, may link T2DM and AD, two amyloid-forming diseases characterized by the existence of fibrillar protein aggregates in brain and pancreas, respectively [62]. Indeed, islet amyloid derived from islet amyloid polypeptide (IAPP), and neurotoxic Aβ can co-deposit in brain and pancreas in both humans and transgenic mouse models, contributing to neuronal loss on one side and peripheral insulin resistance on the other [63,64]. IAPP or amylin, a protein co-expressed and secreted with insulin in β-cells, is associated with β-cells loss, a feature of T2DM pancreatic pathology [65,66]. More recently, IAPP has been implicated in the neurodegenerative process of AD [63,67], as observed in brains from diabetic patients with AD [68]. This is consistent with previous findings reporting an association between degeneration of pancreatic islets and NFTs formation and accumulation [69]. On the same line, recent evidence suggests that inappropriate amounts of AD-related proteins such as Aβ, IAPP, or tau could promote diabetic phenotype, and further exacerbate neurodegeneration [36]. 

Aβ, the main component of senile plaques found in the brains of AD patients, derives from a larger molecule known as the amyloid precursor protein (APP). APP is normally cleaved by members of the α-secretase enzyme family within their extracellular domain. Harmful Aβ amounts are originated when the proteolysis of APP occurs via sequential enzymatic actions of β-site amyloid precursor protein-cleaving enzyme 1 (BACE-1), a β and γ-secretase complex [70]. Afterwards, Aβ protein may undergo additional catabolism by insulin-degrading enzyme (IDE), a metalloprotease enzyme responsible for insulin transformation and the major Aβ degrading enzyme [71]. An imbalance between production, clearance and aggregation of Aβ causes an excessive accumulation of this protein in the brain, triggers AD onset [16] and contributes to the synaptic-toxicity and downstream events that fuel the progression of neurodegenerative diseases [72]. 

The peripheral hyperinsulinemia and insulin resistance under T2DM can accelerate Aβ production by influencing its synthesis, or slowing down its degradation, or impairing both processes. Elevated insulin levels are known to increase the extracellular Aβ levels by modulating γ-secretase activity [73]; furthermore, since insulin and Aβ are both substrates of IDE, the higher insulin concentrations decrease the Aβ clearance by competitively blocking IDE-mediated catabolism [74]. In turn, Aβ oligomers that accumulate under neurodegenerative processes may have a deleterious impact on insulin signaling because, by competing with insulin for IR binding [75], impair the receptor auto-phosphorylation and markedly reduce both IR expression and insulin activities in the dendrites of hippocampal neurons [76,77]. The resulting loss of membrane IRs might therefore represent an early mechanism underlying the memory impairment and other pathological features of AD, and contribute to explain the propensity to develop AD in T2DM patients. 

Overall, these data support the notion that a vicious circle between Aβ pathology and insulin-signaling dysfunction may contribute, among other factors, to synaptic and dendritic spine damage involved in AD pathogenesis [78]. 

### 2.2. GSK-3β an Important Kinase for Insulin-Signaling Pathway and Phosphorylation of Tau Protein

GSK-3β is a multifunctional kinase widely expressed in the brain and involved in a variety of cellular activities including cell development, differentiation and survival [79]. As mentioned previously, the higher expression and dysregulated activity of GSK-3β observed under T2DM might lead to an elevation of Aβ production and an increased phosphorylation of tau protein [80]. On a metabolic site, the serine/threonine kinase GSK-3β acts as downstream target of insulin-mediated PI3K/Akt signaling to help promoting glycogen synthesis and reducing blood glucose levels after a meal [81]. Interestingly, GSK-3β is thought to be constitutively activated by autophosphorylation at Tyr216 and inactivated by phosphorylation at Ser9 [82]. Insulin, by increasing Akt-mediated phosphorylation on Ser9 site of GSK-3β, inhibits its basal enzymatic activity, and modulates the expression of several transcription factors involved in cellular development and life span of neuronal cells. 

On the other hand, phosphorylation on Tyr216 site is positively correlated with the enzymatic activities of GSK-3β. Under conditions that may impair insulin-mediated PI3K/Akt signaling in the brain and therefore decrease the Akt-mediated phosphorylation at Ser9, the persistent phosphorylation on Tyr216 site of GSK-3β may increase tau hyperphosphorylation at residues Ser396, Ser400 and Ser404 [83]. Thus, under impaired insulin signaling, aberrant activation of GSK-3β may result in hyperphosphorylation and accumulation of tau, the main component of NFT and an important determinant for abnormal synaptic plasticity and AD pathophysiology [37,84]. Concomitantly, improper GSK-3β activities may enhance Aβ production and promote Aβ plaque deposition [85]. In turn, Aβ accumulation disrupts GSK-3β activities even more, thus reinforcing the vicious circle that increases tau phosphorylation, impairs ACh synthesis, induces caspase-3 activation and DNA fragmentation in neurons, and sustains microglia-mediated neuroinflammation [38,86]. These multiple and interrelated abnormalities support the neurodegenerative process of dementia and cognitive decline typical of AD and point out to the potential advantages of novel treatment strategies aiming at inhibiting GSK-3β function. 

## 3. Inflammation as One Common Mechanism for Insulin Dysregulation and Neurodegeneration

Inflammation involves both soluble factors and specialized cells that are mobilized to restore normal body physiology [87]. Inflammatory processes are highly involved into the pathogenesis of T2DM as well as neurodegenerative diseases, as provided by clinical and preclinical studies investigating the inflammatory pathways activated in both T2DM and AD [88,89,90]. A chronic state of low-grade systemic inflammation, defined “metaflammation”, is commonly observed under metabolic disorders such as T2DM, obesity and insulin resistance [91], accompanied by an overproduction of peripheral inflammatory cytokines able to cross the BBB and activate brain-resident microglia and astrocytes [92]. In obese patients, fat-derived inflammatory mediators such as TNF-α, IL-1β and IL-6 may be an important addition to cytokines locally produced by CNS-resident microglia [93,94]. 

In addition to Aβ by-products, other peptides generated by enzymatic catabolism may have a role in both DM and AD pathogenesis. Emerging evidence suggest that loss of elastin, a component of extracellular matrix (ECM), may be associated with inflammatory mechanisms underlying vascular aging [95], T2DM [96] and neurological conditions including AD [97]. In the aging brain, matrix metalloproteinases (MMPs) from microglia can degrade elastin and increase the amount of elastin-derived peptides (EDPs). These by-products, in turn, may facilitate the migration of inflammatory cells, and therefore modulate their inflammatory activity with a positive feedback mechanism leading to chronic inflammation. Of note, insulin resistance has been linked to the abnormal expression of neutrophil elastase, a key enzyme for elastin fragmentation; in turn, EDPs have been involved in the development of insulin resistance in mice by a peroxisome proliferator-activated receptor-γ (PPARγ)-dependent pathway [98]. As a member of the nuclear receptor’s family, PPAR-γ controls the cell metabolism of carbohydrates and lipids, and contributes to regulate proliferation, apoptosis, and inflammation. The PPAR-γ- mediated activity is fundamental in vascular and adipose cells, as well as in astrocyte metabolism and in astrocyte-mediated inflammation associated with neurodegenerative diseases [99]. Some pioneering studies exploring the activities of the elastin-derived hexapeptide VGVAPG in astrocytes in vitro suggest that this peptide may increase caspase-1 activity and superoxide dismutase (SOD)-1 protein expression, and simultaneously decrease the release of IL-1β, and the expression of IL-1βR1, catalase (CAT), and NF-kB by a mechanism involving PPAR-γ activation and expression [100]. This mechanism resembles the activity of antidiabetic drugs such as the thiazolidinediones (see next chapters). Although further studies are needed to clarify the peculiar activities of this peptide and its potential anti-inflammatory role, these findings might represent an opportunity to develop new therapeutic strategies in diabetes as well as in neurodegenerative diseases.

Peripheral insulin resistance triggers inflammatory stress signaling in response to cytokines such as TNF-a and IL-6, which activate NF-kB pathways and lead to the transcription of pro-inflammatory genes exacerbating this cycle. In the brain, pro-inflammatory cytokines may then activate cell stress pathways such as the c-Jun NH3-terminal kinase (JNK), the NF-kB signaling IKK complex and RNA-dependent protein kinase (PKR) that, in turn, phosphorylate IRS-1 at serine residues, therefore inhibiting intracellular insulin signaling [101,102,103]. By impairing the protective activities of insulin in the brain, these effects may increase the progression speed of AD development. 

The imbalance between pro- and anti-inflammatory cytokines, able to maintain the inflammatory response at low levels and for a long period of life, is also a feature of AD and aging-related diseases, in which this condition has been termed “inflammaging” [104]. Indeed, elevated concentrations of mediators of the innate immune response and pro-inflammatory cytokines such as IL-6, IL-1β and TNF-α are measured in the brain of AD patients [105]. In animal models of AD and in AD patients the reactive microglia and astrocytes localized around Aβ plaques [106] can be chronically activated. Although these glial cells may reduce Aβ load by phagocytosis, chronic inflammation stimulates the synthesis and secretion of several pro-inflammatory mediators that may therefore exacerbate AD pathology [107]. Interestingly, IL-1 is overexpressed in the brain of AD patients from the initial stages of the disease, and its levels progressively increase with advanced Aβ plaque formation [106]. TNF-α, secreted mainly by microglial cells in response to infection or abnormal aggregation of Aβ oligomers [108], shows increased levels in CSF from AD patients as well as in transgenic models of AD [109,110]. The IRS-1 inhibition subsequent to TNF-α-mediated recruitment of the stress kinases IKK and PKR has been demonstrated in brain of AD patients [111] and in hippocampal neurons of AD animal models [103]. Thus, peripheral insulin resistance in obesity and T2DM and brain insulin resistance in AD show overlapping pathogenic mechanisms that reinforce the hypothesis of a common background [77,103]. 

Consistent with this, markers of peripheral inflammation have been observed in patients with mild cognitive impairment and AD disease [112]. This is in line with results from the Framingham study, suggesting that high serum levels of IL-1β and TNF-α might represent potential biomarkers of AD risk/development [113]. Despite the undisputed contribution of chronic inflammation to both metabolic and neural impairment, it is still unclear whether peripheral inflammation leads to central inflammation or vice versa, and further research is needed to unveil the molecular interplay between T2DM, obesity, and AD.

## 4. Oxidative Stress in Diabetes and Alzheimer’s Disease

Free radicals physiologically generated during cellular activities are directly involved in body metabolism; a small fraction (approximately 5%) of the oxygen produced is converted in reactive oxygen species (ROS), which can act as double-edged sword. Intracellular low levels of ROS are fundamental signaling molecules for a variety of physiological processes, including redox homeostasis and signal transduction [114]. In peripheral tissues, transient ROS generation in response to insulin facilitates insulin signaling, for example by inhibiting protein phosphatases, such as PTEN [115]. In the brain, transient ROS production promotes long-term potentiation and memory-related mechanisms and is involved in synaptic signaling [116]. 

Under pathological conditions, the excessive bioavailability of ROS becomes detrimental for normal cellular signaling. Oxidative stress is consequential to the imbalanced production and accumulation of potentially harmful free radicals, including ROS and reactive nitrogen species (RNS), and insufficient antioxidant neutralizing defense systems. High levels of free radicals (ROS/RNS) can affect biomolecules, including proteins, lipids, and DNA and alter the expression of various stress-response genes, further stimulating additional ROS generation from endogenous sources, compromising cell integrity and leading to cell death [117,118]. Oxidative stress is a central feature in the common pathophysiology of T2DM and AD, and elevated levels of ROS and RNS have been consistently reported in both diabetic and AD patients [119,120,121,122]. 

As diabetes is a life-long disease, the persistent metabolic stress and tissue damage keep fueling the abnormal free radical production over time. Auto-oxidation of glucose, impaired synthesis/function of antioxidant defense enzymes, metabolic abnormalities triggered by hyperglycemia and mitochondrial damage are among molecular mechanisms contributing to oxidative stress under diabetes [122,123]. Superoxide radical (O_2_^−^), perhaps the most renowned among ROS, is mainly produced by cytosolic NADPH oxidase (Nox) activity and by non-enzymatic reaction, which is the main consequence of mitochondrial respiration [122]. Interestingly, increased levels of O_2_^−^, abnormal protein oxidation, and elevated concentrations of thiobarbituric acid reactive species (TBARS) have been found in some brain regions of diabetic animal models [124]. Conversely, the activity of O_2_^−^ scavenging enzymes such as SOD, CAT, or glutathione (GSH) peroxidase, are often decreased [124,125]. On the other hand, in transgenic mice overexpressing APP and lacking antioxidant enzymes, the increased Aβ accumulation suggests that elevated oxidative stress has a great impact on amyloidogenesis [126]. In parallel, studies in humans confirm the evidence that oxidative damage is a feature of the early stages of moderate cognitive impairment, and that levels of oxidized proteins and lipids are elevated in the brain of AD patients compared with healthy controls [127]. Moreover, oxidative stress is involved in increased Aβ deposition in brain of AD patients, with mechanisms related to stimulation of APP-gene expression and reduced activity of α-secretase, which in turn promotes the expression and activation of β and γ-secretases essential for the generation of Aβ from APP [128,129,130,131]. 

Taken together, these data highlight the impact of oxidative stress and the subsequent increased levels of oxidation products in the brain of diabetic animal models as well as in patients with glucose impairment and/or with early features of AD disease.

## 5. Could Mitochondrial Dysfunction Represent the Link between T2DM and AD?

The central role of mitochondria on cell life is explained by their multiple functions on oxidative phosphorylation, energy metabolism and apoptosis [132]. Mitochondria are the major source of ROS, including hydrogen peroxide (H_2_O_2_), hydroxyl (HO^•^) radical and O_2_^−^ that are produced under physiological cellular respiration. Since mitochondria can critically regulate cell survival and death, their abnormal or inefficient activity represents one key feature of cell impairment, including neuronal cell degeneration [30,133]. Of note, because of their limited glycolytic capacity and high energetic needs, neuronal cells are extremely dependent on mitochondria, and therefore critically sensitive to mitochondrial alterations in structure, localization, and function. Thus, mitochondrial dysfunction with subsequent elevated ROS levels might represent a common pathophysiological defect in diabetes mellitus as well as in AD-associated abnormal brain insulin and glucose metabolism. 

The correct synaptic function and transmission between neurons requires normal mitochondrial biogenesis, dynamics, distribution, and trafficking as well as the tight regulation of energy metabolism and calcium availability. Mitochondrial biogenesis requires activation of multiple signaling cascades and transcriptional complexes that promote the formation and assembly of functioning mitochondria. Sirtuins (SIRTs) are a family of nicotinamide adenine dinucleotide (NAD^+^)–dependent protein deacetylases with a key role in mitochondrial biogenesis. Of the seven mammalian sirtuins, the nuclear SIRT1 and the mitochondrial SIRT3 have been linked to neuroprotection in several chronic age-related and aggregate-forming neurodegenerative diseases including AD [134,135]. SIRT-1 deacetylates and activates the transcriptional peroxisome proliferator-activated receptor-*γ* coactivator (PGC)-1*α* that regulates mitochondrial biogenesis and oxidative phosphorylation and contributes to the control of autophagy/mitophagy processes. Activation or overexpression of SIRT1 limits Aβ-dependent toxicity by a mechanism that involves inhibition of NF-kB signaling in microglia [136]. Conversely, impaired SIRT1 activity may disrupt the autophagy/mitophagy quality control, resulting in mitochondrial dysfunction, increased numbers of damaged mitochondria, as well as accumulation of Aβ plaques and tau tangles [137]. The SIRT3 isoform interacts with mitochondrial complex I and, as a downstream target gene of PGC-1*α*, mediates down-regulation of intracellular ROS production dependent on PGC-1α and stimulates mitochondrial biogenesis [138]. SIRT-3 may also protect mitochondria and neurons from excitotoxic and metabolic stress and apoptosis with a mechanism that involves SOD2 and cyclophilin D deacetylation [139]. SIRT1 and SIRT3 can be activated by elevating cellular NAD^+^ levels. Indeed nicotinamide, a precursor of NAD^+^, enhances SIRT3 activity and restores the neuronal mitochondrial bioenergetics, with improved learning and memory deficits in a genetic mouse model of AD. Among other effects, nicotinamide increases mitochondrial resistance to oxidative stress, enhances PI3K/Akt and MAPK/ERK1/2 signaling pathways, and promotes the transcription factor CREB by SIRT1 [140]. 

Perturbations in dynamic properties of mitochondria, including fission, fusion, motility, and turnover [141] contribute to oxidative stress, synaptic damage, and neurodegeneration, which are among pathological features in the brain of diabetic subjects and may help to explain their impairment in cognitive abilities [142]. Compared to other body tissues, the brain is more susceptible to oxidative imbalance due to its high energy demand, high oxygen consumption, rich lipid content and paucity of antioxidant enzymes [143]. In the brain, approximately 90% of oxygen-dependent ATP required for neuronal function is provided by a complex organization of proteins in the mitochondrial electron transport chain (ETC) working in team to exert a process known as oxidative phosphorylation. Thus, any impairment of oxidative phosphorylation due to mitochondrial dysfunction affects the CNS earlier than any other system: by decreasing the amount of ATP necessary for the transmission of impulses along the neural pathway, abnormal oxidative phosphorylation may therefore contribute to failure in neuronal metabolic control and facilitate neurodegeneration [144]. In brain regions of AD patients, the abnormal mitochondrial structure and function correlates with changes in glucose metabolism and oxygen consumption [145], as well as with impaired activity of the ETC enzyme complexes including the cytochrome oxidases [146]. The improper function of damaged mitochondria, which results in higher production of ROS but decreased synthesis of ATP, might therefore trigger a vicious circle by increasing the oxidative damage of proteins, carbohydrates, and lipids, which in turn contribute to the amplified generation of ROS, critical components for the pathogenesis of AD [127]. Moreover, a decreased cerebral metabolism may also result from impaired activity of key enzymes involved in tricarboxylic acid cycle (TCA) such as isocitrate dehydrogenase, pyruvate dehydrogenase and α-ketoglutarate dehydrogenase complexes, as observed in fibroblasts and postmortem brain tissue from AD patients [147]. These enzymes are highly susceptible to oxidative modification and their activities may change under exposure to pro-oxidant conditions [148]. The ROS-induced abnormal function of TCA enzymes, in turn, may further impair the efficiency of mitochondrial energy-related proteins and correlates with the clinical progression of mental disturbances in AD patients, suggesting a coordinated mitochondrial alteration [147].

Whether mitochondrial dysfunction is the main cause of AD or occurs because of AD pathogenesis is still an open question. In a variety of studies investigating the pathological changes under aging and AD, synaptic mitochondria have been indicated as reservoir for Aβ build-up [149,150]. Accumulation of Aβ in mitochondria causes mitochondrial swelling, ROS overproduction, impaired respiratory chain function [151,152] and altered calcium homeostasis [153,154], with subsequent further damage of mitochondrial structure, inhibition of ATP production, and defective energy metabolism. These findings suggest that Aβ aggregation in mitochondria precedes the subsequent, age-related, extracellular Aβ deposition responsible for synaptic damage in AD brains; thus, according to this hypothesis, Aβ accumulation in mitochondria may represent the initial pathological event triggering mitochondrial perturbations, which in turn contribute to neurodegeneration [149]. 

In contrast, the hypothesis of a "primary mitochondrial cascade" as the main insult underlying the pathophysiology of late-onset AD is based on the concept that AD is a multifactorial disease, and not just a linear downstream consequence of Aβ deposition [155]. Consistent with this view, the brain neurodegeneration observed in patients with AD would result from mitochondrial failure, which compromises the production of cellular energy and, by losing the ability to buffer intracellular calcium and causing the opening of the mitochondrial permeability transition pore (mPTP), leads to release of harmful ROS. Uncontrolled oxidative stress triggers the discharge of cytochrome C and activates the apoptotic cascade, contributing to the progressive decline in long-lived neuronal cells and memory impairment [29,156]. Moreover, in cells with defective mitochondria, the imbalanced activity of ROS scavenging systems may worsen the deleterious consequences of high ROS levels.

An increasing body of evidence supports the view that the decline in mitochondrial function is a common defect shared among age-related diseases, including AD and T2DM [157]. Under diabetes, brain changes linked with neurodegeneration and cognitive decline, such as elevated tau expression/phosphorylation and Aβ accumulation [10,158], synapses loss, impaired learning and memory, synaptic plasticity deficits, [28,159,160], have been repeatedly observed in conjunction with oxidative stress [161,162], disruption of mitochondrial dynamics, and mitochondrial dysfunction [26,27,162]. Similarly, an age-related impairment of the respiratory chain and uncoupling of oxidative phosphorylation has been detected in brain mitochondria of animal models, where mitochondrial dysfunction precedes Aβ aggregation and likely contributes to pathological molecular cascades mediating or initiating AD-like disturbances [163,164]. As mentioned above, the role of amyloid in mitochondrial dysfunction and ROS production suggests that Aβ directly induces oxidative stress, with a subsequently impaired insulin signaling in the peripheral tissues [165,166]. As in neurons, a proper mitochondrial function is fundamental for insulin secretion from β-cells, as it completely depends on ATP generation [167]. Several mitochondrial-related abnormalities, such as lower mitochondrial mass [168], altered mitochondrial morphology [169], reduced fatty acid oxidation [170], overproduction of ROS with ATP depletion, and decreased antioxidant abilities [171,172], have been observed in human and animal models of T2DM. In adipocytes and muscle cells, increased ROS production and/or decreased antioxidant capability modifies phosphorylation of insulin signaling and promotes the activation of stress kinases [173]; on the other hand, inhibition of mitochondrial dynamics and increased lipid peroxidation have been described in skeletal muscle of patients with insulin resistance [168]. 

**Figure 2:**
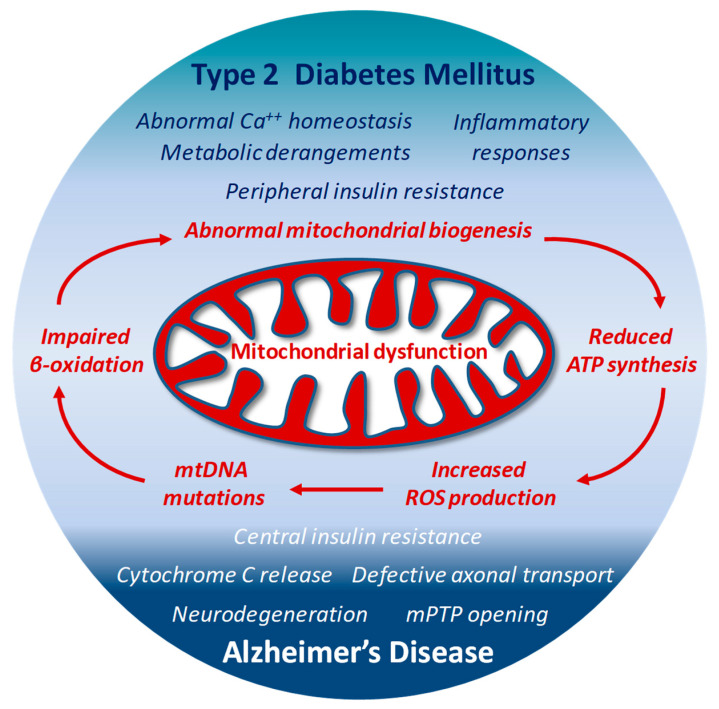
Structural and functional alterations in mitochondria by either defective insulin signaling, or neurodegenerative mechanisms may represent a connecting point between T2DM and AD-associated abnormal brain insulin metabolism.

The high ROS levels generated by dysfunctional mitochondria negatively affect specific mitochondrial components, including membrane lipids, specific enzymes of ETC and even mitochondrial DNA (mtDNA) [127,155]. The close proximity to the electron transport machinery, and the lack of histones makes mtDNA particularly vulnerable to ROS oxidative reactions. The 13 proteins encoded by mtDNA are all essential for proper functioning of the ETC and for the mitochondrial homeostasis itself [174]. Thus, when mtDNA is damaged and its transcription impaired, the function of the ETC is further jeopardized and ROS production exacerbated [175], with subsequent deeper changes in dynamics (fission and fusion) and biological functions of mitochondria, and increased risk of additional mutations in mtDNA. This last condition has been reported in patients with T2DM, in which the presence of mtDNA mutations correlates with impaired synthesis of mitochondrial proteins and down-regulation of both mitochondrial function and gene expression [176]. In animal models, the lack or deletion of mitochondrial genes in pancreatic β-cells results in impaired oxidative phosphorylation, and triggers diabetes [177]. Interestingly, mtDNA mutations responsible for ETC abnormalities and impaired ATP production profoundly affect brain function and may facilitate the onset of AD [156]. Under conditions characterized by the systemic increase of oxidative stress, mtDNA mutations and reduced transcription levels of crucial proteins have been found in blood samples from AD patients [178] (Figure 2)

Thus, mitochondria are central coordinators of energy metabolism, and concomitantly sources and targets of ROS; their structural and functional alterations by either defective insulin signaling or neurodegenerative mechanisms may represent a connecting point between T2DM and AD-associated abnormal brain insulin metabolism: on one side, Aβ accumulation and tau hyperphosphorylation synergistically alter mitochondrial bioenergetics and exacerbate oxidative stress, which accelerates neurodegenerative progression. On the other side, dysfunctional insulin signaling associated with a reduced cerebral energy metabolism makes neurons more vulnerable to ROS harmful effects, advancing mitochondrial dysfunction and worsening oxidative stress. Although a cause/effect relationship is hard to draw, the vicious circle between defective insulin signaling, increased deposition of Aβ, impaired metabolic homeostasis and oxidative stress points to mitochondrial dysfunction as one of the most important underlying propellants for both T2DM and AD pathogenesis.

## 6. Insights from Treatment Approaches for Both AD and T2DM

For more than 30 years, research centered on the “amyloid cascade hypothesis” [179], has resulted in unsuccessful attempts to develop effective drugs for AD patients [23,180]. Thus, at present, the management of AD is only symptomatic. Of the four drugs currently prescribed, three of them (donepezil, rivastigmine and galantamine) are inhibitors of acetylcholinesterase (AChE), while memantine is a N-methyl-D-aspartate (NMDA) receptor antagonist. Unfortunately, these drugs show only a modest effectiveness in improving the cognitive ability of patients with mild/moderate AD; furthermore, they do not prevent neuronal loss, or brain atrophy, nor the progressive deterioration of cognitive processes. Following its accelerated FDA approval in 2021 (https://www.fda.gov/drugs/postmarket-drug-safety-information-patients-and-providers/aducanumab-marketed-aduhelm-information, 7 June 2021) aducanumab, a human monoclonal antibody, has become the first novel therapy for AD since 2003. Although aducanumab has been shown to dose- and time-dependently reduce brain Aβ plaques in patients with prodromal or mild AD, its real effectiveness on clinical symptoms and progressive decline is still a matter of debate [181,182,183] and the search for effective drug treatment is far from being complete. An extensive review of the most relevant and novel pharmacological approaches for AD is beyond the purpose of this paper and can be found elsewhere [184,185,186]. 

### 6.1. Ketogenic Diet (KD)

Among non-pharmacological treatments, ketogenic diet (KD) has been proposed as a novel metabolic treatment in various diseases. The KD increases fat and reduces carbohydrate consumption, decreasing insulin and stimulating liver oxidation of fatty acids to ketone bodies (β-hydroxybutyrate, acetoacetate and acetone) that enter the bloodstream and are available to brain, muscle, and heart, where they generate energy for cells in the mitochondria. Preclinical findings in transgenic mice models of AD suggest that administration of elevated levels of β-hydroxybutyrate (BHB) reduces brain Aβ levels, protects from amyloid β-toxicity and improves mitochondrial function [187], with mechanisms likely related to the BHB-mediated increased expression of brain derived neurotrophic factor (BDNF) and resulting beneficial effects on cell metabolism and mitochondrial biogenesis [188]. In a pilot study evaluating KD in AD patients, an improvement of cognitive performance was observed [189]; however, the small size and single-arm structure of the study do not allow any definitive conclusion on the beneficial effects of this treatment. The best results are observed in early pre-symptomatic stages of AD and the improvement of cognitive outcomes depends on the level and duration of ketosis. Data available so far suggest that KD may improve cognition in AD patients, but results are strictly associated with several factors including the stage of AD, its progression, or the ApoE4 genotype [190]. Hopefully, results from other clinical studies will broaden our understanding in this field

### 6.2. Antidiabetic Drugs

The proposed causative link between insulin-signaling dysfunction and pathogenic mechanisms in the AD brain has provided a rationale for “drug-repositioning” strategies, and the effects of antidiabetic drugs have been investigated in patients with both T2DM and AD [191]. Preclinical studies in animal models have extensively demonstrated that a correct insulin signaling contributes to long-term memory consolidation and improves spatial learning [192,193,194], and that insulin regulates neuronal survival by activating either its own receptor or IGF receptors [195]. Based on the assumption that the structural and functional brain alterations responsible for cognitive deficits in the elderly are related to an impaired insulin sensitivity [196], therapeutic approaches to restore brain insulin signaling could be beneficial in age-related neurological diseases. Consistent with this idea, and in line with the high density of IRs in the hippocampus—a brain region associated with cognitive functions—intranasal insulin administration has been shown to enhance verbal memory in patients with mild cognitive impairment and late-onset AD, with a concomitant improved cerebral glucose metabolism in those brain regions affected by AD changes [191,197]. In contrast to hypoglycemic episodes and systemic insulin resistance that strongly limit intravenous insulin use, both acute and long-term intranasal insulin administration have shown beneficial effects on cognitive functions with only minor side effects such as mild rhinitis [198]. Despite encouraging results, however, the role of insulin administration on cognitive decline is still controversial. The variable therapeutic results of acute insulin administration on CNS may depend on APOE genotype, a strong genetic predictor for AD [199,200]. 

With the availability of glucagon-like peptide-1 (GLP-1) analogues, these drugs have been proposed as alternative therapeutic approach, or in addition to insulin-based therapies, in AD patients. The intestinal GLP-1 is involved in glucose homeostasis [201] but shows some interesting neuroprotective effects, as observed in the brain of AD mice models whose hippocampal neurons are protected from oxidative stress and Aβ-mediated harmful effects on synaptic plasticity. Like endogenous GLP-1, GLP-1 receptor agonists (GLP-1RA) cross the BBB and bind receptors widely expressed in the frontal cortex, hypothalamus, thalamus, hippocampus, cerebellum, and substantia nigra. Exenatide-4, liraglutide and lixisenatide have all been investigated as potential treatments in AD [191]. Exenatide-4 decreases AD-associated tau protein hyperphosphorylation in the hippocampus of T2DM rats and, by favoring activation of PI3K/Akt and deactivation of GSK-3β signaling [202] improves the dysfunctional insulin pathway in the brain. These findings are in line with previous investigations on exenatide-4, linking the reduced IRS-1 phosphorylation level on serine residues and the activated JNK pathway to the improved cognitive functions [77]. Similarly, in AD mice models, administration with liraglutide prevents chronic inflammation, reduces neuronal tau hyperphosphorylation, enhances synaptic plasticity, decreases the formation of β-amyloid deposits in the brain [61] with a concomitant amelioration of PI3K/Akt signaling pathway and improved memory impairment [203]. The neuroprotective activities of lixisenatide, a long-lasting GLP-1 RA, have been related to the activation of Akt-MEK1/2 signaling pathways and the regulation of calcium homeostasis [25]. Although still too preliminary to draw definitive conclusions, the evidence that GLP-1 RA exert influence on AD pathology by multiple mechanisms is compelling. Results expected from the ELAD study, testing the effect of liraglutide in patients with AD (clinicaltrials.gov NCT 01843075) [204] will help to clarify whether GLP-1 analogues represent a class of drugs potentially important for AD treatment.

Thiazolidinediones (TZDs), approved as a glucose-lowering therapy for patients with T2DM, target the PPARγ and are among antidiabetic drugs evaluated for their potential role in AD pathophysiology. In addition to a positive effect on insulin resistance and insulin signaling, TZDs display neuroprotective effects in AD mainly secondary to the inhibition of inflammatory gene expression and decreased Aβ generation and deposition [205,206]. In initial studies, AD patients treated for 4 to 6 months with rosiglitazone showed an improvement of memory performance and selective attention compared with control subjects [200]; similarly, in a genetically defined population with mild-to-moderate AD, a 6-month treatment with rosiglitazone produced a significant improvement in cognitive performance [207,208]. Despite promising results, subsequent clinical trials with larger numbers of patients and a longer duration of treatment achieved poor results [209,210]. With rosiglitazone withdrawal from the market due to increased cardiovascular risk [211], the only TZD presently available is pioglitazone, able to reach the brain and control glial activation in AD-related pathologies [212]. In mouse models of AD, treatment with pioglitazone for 4 months enhanced the Akt signaling, attenuated tau hyperphosphorylation and neuroinflammation, and concomitantly improved learning abilities [213]. In diabetic patients with mild AD, results are controversial: in a network meta-analysis of several clinical trials, pioglitazone improves verbal memory, general cognition and regional cerebral blood flow compared to placebo [214,215,216,217]. However, other clinical studies failed to demonstrate pioglitazone efficacy [218,219]. Results expected from TOMORROW clinical trial (ClinicalTrials.gov NCT01931566), started in 2013 and completed in September 2019, will hopefully help to shed light on the efficacy of low-dose pioglitazone to delay the onset of mild cognitive impairment in normal individuals at high risk of AD.

### 6.3. GSK-3β Inhibitors

Based on the observation that GSK-3β overexpression/overactivation in diabetic patients doubles their risk to develop AD [220], many GSK-3β inhibitors have been synthesized, and some of them evaluated in clinical studies as drugs for neurological diseases and AD treatment [221,222]. With respect to GSK-3β inhibitors binding on the ATP site, the class of non-ATP competitive molecules displays a higher selectivity and a lower toxicity, and is therefore considered more promising for therapeutic aims. In transgenic mice overexpressing human mutant Aβ PP and tau protein, tideglusib, an irreversible non-ATP-competitive GSK-3β inhibitor [223], promotes reduction of tau phosphorylation levels and brain Aβ deposition, and prevents hippocampal neuronal cell death and memory loss [224]. In a randomized trial, tideglusib has been shown to slow down atrophy progression in the whole brain; unfortunately, despite the compound safety, results from completed Phase II trials did not measure any significant clinical efficacy in patients with mild-to-moderate AD [222]. However, since tideglusib is also a PPARγ-receptor agonist, its double mechanism suggests that tideglusib-like compounds may display protective effects in patients with both diabetic and neurodegenerative processes [225]. Indeed, in diabetic db/db mice, intrahippocampal infusion of TDZD-8 (another non-ATP competitive thiazolidinedione inhibitor of GSK-3β) counteracts tau hyperphosphorylation and normalizes hippocampus-dependent memory, further supporting a role for GSK-3β inhibition in protecting from T2DM-induced memory impairment [226]. 

### 6.4. Antioxidant Compounds

The wide number of “antioxidants” evaluated as potential preventative strategies in diabetes and AD emphasizes the crucial role attributed to redox imbalance in the pathogenesis of both diseases [227,228]. Most of these molecules are natural compounds such as polyphenols, known to attenuate the ROS and RNS levels and counteract the increased production of advanced glycation end products (AGEs) at the sites of inflammation [229]. Furthermore, by sequestering ROS and RNS, they prevent the formation of toxic Aβ oligomers and modulate tau protein hyperphosphorylation and NFTs formation [230]. However, several other mechanisms may contribute to explain the overall effects of these molecules. For example, resveratrol, one of the most renowned polyphenols, exerts neuroprotective effect by remodeling Aβ soluble oligomers and fibrils into nontoxic aggregates [231], and regulates NF-kβ signaling pathways by activating the SIRT1 deacetylases, with subsequent mitigating effects on neuronal degeneration and inflammaging [232,233]. The resveratrol-mediated increase in SIRT1 expression, and the concomitant activation of both AMPK and PGC-1α contribute to explain the beneficial effects on mitochondrial biogenesis, whose physiological function is to reduce the production of superoxide radicals by increasing the activity of complexes III and protecting against oxidative stress [234]. The resveratrol-dependent activation of SIRT1 in neurons prevents Aβ-induced microglial death and contributes to improved cognitive function. Moreover, SIRT1 activity by resveratrol decreases the content of pro-inflammatory cytokines IL-1β and IL-18, and up-regulates the antioxidant defenses by increasing SOD and GSH content [234]. Concomitantly, the resveratrol-mediated inhibition of IAPP cytotoxic aggregates [235], and its ability to reduce hepatic receptor for advanced glycation end products (RAGE) expression, to decrease glucose plasma levels and to increase peripheral insulin sensitivity in T2DM rodents [236,237] has prompted several clinical trials aiming at exploring its protective effects on glucose-intolerant or diabetic subjects [238]. Moreover, following the observation that resveratrol-mediated AMPK activation triggers autophagy and lysosomal degradation of Aβ in models of AD [239,240], its effects have been evaluated on cognitive function of AD patients. Results from two studies on mild-to-moderate AD patients have shown uncertain results [241,242]. An ongoing clinical trial (NCT 02502253 clinicaltrials.gov) will assess the reduction of brain Aβ, tau burden and blood glucose levels in AD patients receiving a bioactive dietary polyphenolic preparation (BDPP), which has demonstrated to improve cognition and brain plasticity long-term potentiation (LTP) in mouse models of metabolic syndrome and AD. The study will also be crucial to demonstrate the CSF penetration of oral BDPP and evaluate the effects in patients with mild cognitive impairment (MCI) and T2DM. 

Curcumin, a brain permeable compound, is another example of natural antioxidants with neuroprotective effects in AD mouse model [243], able to reduce the formation of human IAPP amyloid fibrils [244] and to inhibit GSK-3β activities [61,245]. Currently, two clinical trials are in progress to evaluate curcumin effects in T2DM patients (clinicaltrials.gov NCT02529982; clinicaltrials.gov NCT04528212). The limited efficacy of curcumin in clinical studies on AD patients [246,247] could be explained, at least in part, by its poor plasma solubility and subsequent low bioavailability [248]. 

The current research on antioxidants suggests that several other natural compounds such as zerumbone, gingko biloba, capsaicin and lycopene may possess interesting properties to fight the pathogenesis of both T2DM and AD pathology as specified in Table 1 (reviewed in [61]). For most of them, an additional challenge is represented by the specific pharmacokinetic profile and bioavailability in humans.

### 6.5. Mitochondria-Targeted Drugs

**Figure 3:**
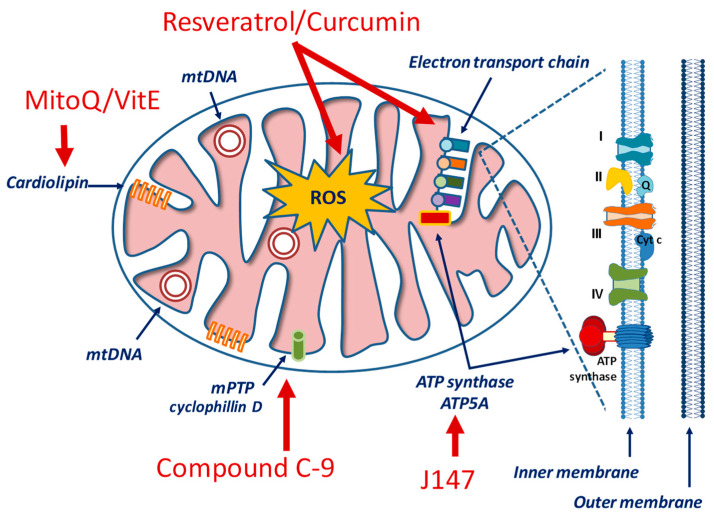
Simplified view of mitochondrial structure and examples of drugs targeting specific components.

Considering the critical standpoint of mitochondria in cellular processes, the design of mitochondria-specific targeting approaches represents a current trend in molecular pharmacology for cardiovascular, neurological, inflammatory, metabolic and hyperproliferative conditions [249,250,251]. Drugs aiming at regulating either mitochondrial bioenergetics (as, for example, glucose metabolism and/or the ETC) or mitochondrial homeostasis (that involves mitophagy and mitochondrial biogenesis) should take into account some unique features of these organelles, including the high transmembrane potential (ΔΨm) across the inner mitochondrial membrane (IMM), a distinctive phospholipid composition (represented by cardiolipin) in the IMM, and a specific protein import machinery with a special amino acid sequence [252,253] (Figure 3). 

In the context of AD pathology, therapeutic candidates include the J147 compound that exerts a regulatory role on the AMPK/mTOR signaling pathway, a canonical longevity signaling [254]. The mTOR is a serine/threonine protein kinase whose activities modulate a wide variety of cellular signals related to cell growth, motility, proliferation, and survival, as well as protein synthesis and transcription. mTOR inhibits autophagy and this effect has critical consequences under Aβ and tau protein dysregulation. mTOR is under AMPK control and it is inhibited when AMPK is phosphorylated and activated. Consequently, while autophagy is inhibited by mTOR, it is promoted by AMPK. Emerging studies show that increase in cytosolic Ca^2+^, via the calcium/calmodulin-dependent protein kinase kinase β (CAMKK2)-mediated activation of AMPK, restores autophagy -by inhibiting mTOR- and promotes lysosomal degradation of Aβ in AD [254]. By targeting the α-F1 subunit of ATP synthase (ATP5A), J147 compound causes a sustained CAMKK2-dependent phosphorylation of AMPK at Thr172, prolongs mTOR inhibition and dampens ATP expenditure, therefore increasing autophagy. A phase I clinical study is currently ongoing to assess the safety profile and PK properties of J147 in healthy subjects (ClinicalTrials.gov Identifier: NCT03838185).

Rapamycin (a potent and selective mTOR inhibitor), latrepirdine and nicotinamide are among compounds currently studied as regulators of the autophagy pathway for their potential use in AD [233]. Latrepirdine, an antihistaminic drug, can reduce defects of mitochondria and Aβ toxicity by regulating the autophagic pathway [255]. In a Phase II clinical trial on AD patients, latrepirdine has been shown to significantly improve cognitive function [256]. Nicotinamide, the precursor of nicotinamide dinucleotide (NAD^+^), reduces Aβ and tau pathologies via various activities that increase brain bioenergetics, mitochondrial response to oxidative stress and autophagy [257].

Thiamet G is a potent and specific inhibitor of O-GlcNAcase, an enzyme that removes N-acetylglucosamine from glycoproteins, and has been shown to reduce Aβ and tau pathology and to rescue cognitive deficits in mouse models of AD [258]. MitoQ and SkQ1 are the derivatives of ubiquinone and plastoquinone, respectively. In addition to their uncoupling activity, these compounds can bind to mitochondrial cardiolipin and prevent its oxidation. To improve mitochondrial localization, mitoQ [259] and mitoVitE [260] have been developed. They contain coenzyme Q and vitamin E, but have also a lipophilic cation that can penetrate the BBB and localize into mitochondria. Similarly, various small peptide antioxidants have been developed to improve cellular penetration and mitochondrial localization [261]. Although some of these compounds are ineffective to treat AD patients [262], other clinical trials are ongoing to ascertain their effects on other mitochondrial-based diseases [253]. 

Compounds targeting mitochondrial activities and acting as division inhibitors [263] and mitophagy activators [264] are relatively novel approaches with a potential to enhance cognitive impairment in animal models of AD [265,266]. Mitophagy allows damaged mitochondria to be selectively identified, ubiquitined, and degraded. Under this process, abnormal mitochondria are sequestered to form autophagosomes and subsequently delivered to lysosomes for degradation. This mechanism is essential in long-lived cells such as neurons, where mitophagy is imperative for tissue maintenance and cellular homeostasis. The importance of mitophagy in the tight regulation of mitochondrial quality control suggests its potential role as target for therapeutic strategies in AD [267]. A recent study shows that enhancing mitophagy may prevent important AD features, including cognitive impairment, tau hyperphosphorylation, Aβ accumulation and neuroinflammation, highlighting the importance of mitochondrial quality control in therapeutic intervention of AD [239].

Since the suppression of the mPTP opening has been suggested to restore the structural and functional integrity of mitochondria in AD neurons, modulators of cyclophilin D (CypD), the most well-characterized component of mPTP, have also been evaluated to improve mitochondrial dysfunction in animal models of AD [268]. 

Although the clinical translation from animal models to human pathology is critical, and the lack of reliable biomarkers as well as the long-term progression of both AD and T2DM add complexity to the studies, compounds acting on restoration of mitochondrial function in combination with current available treatments may provide an additional therapeutic option in slowing down the progression of AD-related disturbances in diabetic subjects.

## 7. Therapeutic Perspectives and Conclusions

Although the search for novel therapeutic strategies in T2DM has permitted the availability of several new drugs, identifying successful treatments for AD is a task not yet completely resolved. The multifactorial nature of AD, its long-term progression, the difficult translation of results from AD animal models to clinical pathology, the lack of reliable biomarkers is among critical factors still halting this goal. Moreover, the brain tissue-specificity and the inability of many therapeutic agents to cross the BBB must be considered when planning effective AD therapy. In addition, observations from several clinical trials suggest that targeting a single pathological feature of AD pathophysiology may not give the expected therapeutic outcomes.

Mitochondrial dysfunction has been associated with the pathophysiology of many disorders, including diabetes and neurodegenerative diseases. Besides genetic defects in which mitochondrial dysfunction could represent the culprit, its role as a disease-causing mechanism is still a matter of debate. In diseases with complex etiology, it may represent a secondary phenomenon. Nevertheless, the identification of mitochondrial dysfunction as common background of diabetes and AD-induced neurodegeneration might help our understanding of diseases mechanisms, potentially leading to novel therapeutic avenues. Thus, strategies settled to counteract diabetes-induced cognitive impairment and AD-mediated neurodegeneration encompassing mitochondrial dysfunction and redox status imbalance will hopefully broaden the therapeutic options currently available for these two progressive and often correlated diseases. On this ground, the availability of “mitochondrial medicine” that can restore mitochondrial function and mitochondrial bioenergetic pathways in the brain can be foreseen as a novel opportunity for therapeutic perspectives aiming not only to delay, prevent, or treat age- and metabolic-related diseases, but also to provide additional options in combination with currently available treatments.

## Figures and Tables

**Table 1:** Drugs used for treatment of Type 2 Diabetes Mellitus (T2DM) and Alzheimer’s Disease (AD).

T2DM	Drugs	AD
↑ glucose uptake and regulation	**INSULIN**	↑ deactivation of GSK-3β ↑ Aβ clearance
↑ insulin secretion	**GLP1-RA** (Exenatide-4, liraglutide, lixisenatide)	↑ deactivation of GSK-3β ↓ neuronal Tau hyperphoshorylation
↑ insulin sensitivity, ↑ transcription of insulin sensitive genes	**TZDs** (rosiglitazone, pioglitazone)	↓ Aβ generation and deposition
↑ insulin sensitivity	**GSK-3β INHIBITORS** (Tideglusib, NP12, TDZD-8)	↑ deactivation of GSK-3β ↓ Tau hyperphosh ↓ Aβ deposition
↓ RAGE expression ↑ glucose uptake ↑ insulin sensitivity	**RESVERATROL**	↑ AMPK ↓ mTOR ↑autophagy ↓ Aβ deposition
↑ insulin sensitivity ↓ glucose plasma levels	**CURCUMIN**	↓ IAPP amyloid fibrils ↑ deactivation of GSK-3β
↑ insulin sensitivity	**ZERUMBONE**	AChE inhibitor
↓ intestinal glucose absorption	**CAPSAICIN**	↓ RAGE activation ↓ blood–brain Aβ
↓ free radicals	**LYCOPENE**	↓ free radicals
	**J147**	↑ AMPK ↓ mTOR ↑autophagy ↓ Aβ deposition
	**THIAMET G**	O-GlcNAcase inhibitor ↓ Aβ and Tau pathology
	**MITOCHONDRIA ANTIOXIDANTS** (MitoQ and MitoVitE)	↓ free radicals ↓ Aβ neurotoxicity
	**RAPAMYCIN**	↓ mTOR ↑autophagy/mitophagy
	**LATREPIRDINE**	↑autophagy/mitophagy ↓Aβ toxicity
	**NICOTINAMIDE**	↑ autophagy ↓Aβ and Tau pathology.

Abbreviations: GLP-1 RA: glucagon-like peptide-1 receptor agonists; TZDs: thiazolidinediones; AChE: Acetylcholinesterase; RAGE: Receptor advanced glycation end products. ↑ increase, ↓ decrease.

## Data Availability

Data is contained within the article.
